# A systematic review of dramatherapy interventions used to alleviate emotional distress and support the well‐being of children and young people aged 8–18 years old

**DOI:** 10.1002/jcv2.12145

**Published:** 2023-03-01

**Authors:** Eleanor Keiller, Megan Tjasink, Jane Bourne, Dennis Ougrin, Catherine Elizabeth Carr, Jennifer Y. F. Lau

**Affiliations:** ^1^ Queen Mary University of London London UK; ^2^ Queen Mary University of London & Barts Health NHS Trust London UK; ^3^ Cumbria Northumberland Tyne and Wear NHS Foundation Trust London UK; ^4^ East London NHS Foundation Trust & Queen Mary University of London London UK

**Keywords:** anxiety, arts therapies, depression, dramatherapy, emotional distress, mental health, trauma

## Abstract

**Background:**

Dramatherapy, a creative form of psychotherapy, may be a useful treatment for child and adolescent mental health. As there is a growing evidence base, this systematic review sought to identify, describe and evaluate dramatherapy with children and adolescents who were experiencing emotional distress (anxiety, depression and trauma) in order to inform future research in this area.

**Methods:**

Seven databases (PsychInfo, PubMed, Scopus, Web of Science, CINAHL, EMBASE and Cochrane) were searched for peer‐reviewed articles exploring dramatherapy as a treatment for child and adolescent emotional distress. Hand searches of relevant journals were also conducted. Two reviewers coded articles for eligibility and independently appraised papers using the Joanna Briggs Institute Critical Appraisal Tools. Details relating to intervention and participant characteristics were extracted and, where data were available, effect sizes on measures relating to emotional distress were calculated.

**Results:**

Fifteen papers were included. Studies showed that dramatherapy was often delivered in schools (46%) and clinical settings (20%) and was more frequently delivered to adolescents (53%) (>11 years) than children (26%) (8–11 years). Dramatherapy was used as a treatment for diagnostically heterogeneous groups (40%), for emotional and behavioural difficulties (33%) and following a shared, traumatic, experience (20%). Seven papers reported relevant quantitative data however, just three of these studies were controlled and none were blinded. Pre‐to‐post intervention effect sizes ranged from *d* = 0.17 to *d* > 2 yet samples were small and participant response to treatment was not always consistent. The largest effects were seen in dramatherapy employed following trauma and in clinical settings. Medium to large effects were also seen in early intervention school‐based dramatherapy.

**Conclusion:**

Despite promising results with regards to the treatment of child and adolescent emotional distress, the evidence base for dramatherapy is small and methodologically flawed. Larger, methodologically robust trials should test the efficacy of dramatherapy in future research.

## Graphical abstract

Dramatherapy, a creative form of psychotherapy, may be a useful treatment for child and adolescent mental health. As such, this systematic review sought to identify, describe and evaluate dramatherapy which is used as a treatment for emotional distress. Results suggest that dramatherapy provision is varied, however, large effects were seen in dramatherapy employed following trauma and in clinical settings; medium to large effects were also seen in early intervention school‐based dramatherapy.

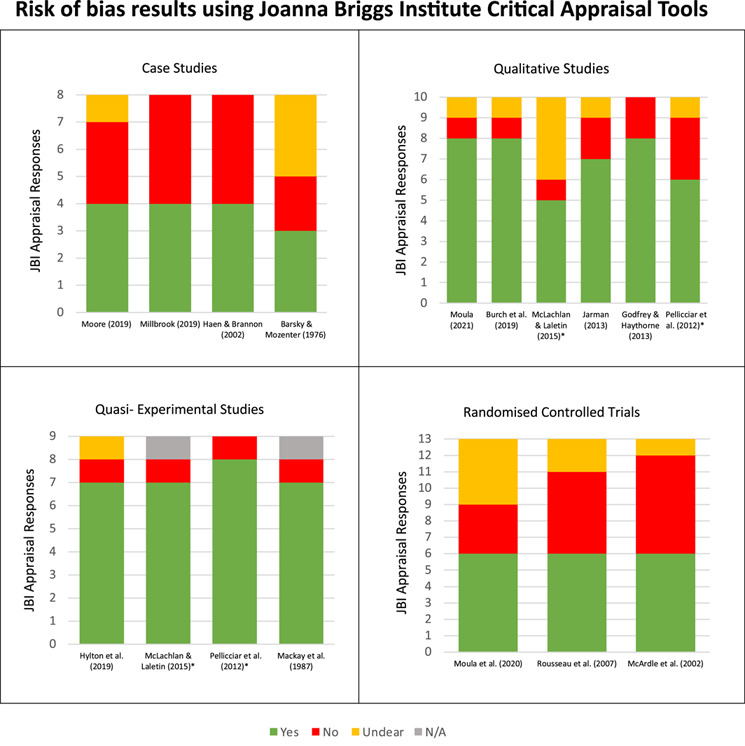

**Key points**

- The evidence base of dramatherapy (a creative form of psychotherapy employing aspects of drama to facilitate the therapeutic process) is small but growing.
- Dramatherapy is currently used with CYPs as a treatment for a range of conditions related to emotional distress including anxiety, depression and trauma.
- Provision of dramatherapy is largely heterogeneous but much work takes place in schools (46%) and with adolescents (53%).
- Existing research is methodologically limited. Among other issues, studies are small and frequently uncontrolled.
- Dramatherapy may be useful for high‐level clinical needs or following a traumatic event; it may also be useful for early intervention however further research, focused on methodological quality, is required.

## INTRODUCTION

Anxiety and depression are leading causes of illness and disability; they affect millions globally each year and cause significant social and economic impacts (Pote, 2022). Along with post‐traumatic stress disorder, these conditions are primarily characterised by internalised mental anguish and can be given the umbrella term: emotional distress. Although they occur across the lifespan, these conditions commonly arise before or during adolescence (Jones, 2013; Solmi et al., 2022). The personal and social burden of experiencing an emotional disorder during childhood and adolescence is significant. The impact can be felt across many facets of life including via missed school days (Finning et al., 2019; NHS Digital, 2021), impaired social relationships (Elmer & Stadtfeld, 2020) and via economic cost (Konnopka & König, 2020). Intervening to treat emotional distress during childhood and adolescence is critical to reducing long‐term human suffering and significant economic burden.

A standard, often frontline treatment for conditions characterised by emotional distress is cognitive behavioural therapy (CBT) (Saavedra et al., 2010; Yang et al., 2017) and other psychological therapies such as psychoanalysis (Göttken et al., 2014; Silver et al., 2013). Although CBT is a well‐established treatment, children and young people (CYPs) often face lengthy waiting lists and some research suggests that CBT may be no more effective than placebo (Ale et al., 2015). An additional challenge posed by CBT, and other psychological therapies, is the individual differences in treatment response (Hudson et al., 2015); while some children respond well, others may worsen or experience no change. An alternative treatment is psychotropic medication, however, this is not recommended for all CYPs (National Institute for Health and Care Excellence, 2019) and the low acceptability of this option for some families (Gibson et al., 2014; Radez et al., 2021) means that many CYPs go without treatment (World Health Organisation, 2021). The ever‐increasing need for youth mental health services, and the increasing understanding that “one size does not fit all”, means that there is a significant need to explore alternative and effective mental health interventions which offer treatment choices to this population group.

Recently, an area of great interest in health research has been the arts. A detailed scoping review, published by the WHO in 2019, outlines many areas wherein the arts can positively impact upon health. From using singing toothbrushes to improve dental care to using dance to aid communication in people with dementia, the potential for the arts to improve health is vast (Fancourt & Finn, 2019). Relating more specifically to child and adolescent mental health, a scoping review by the Wellcome trust found that, arts‐based interventions led to significant decreases in anxiety and depression symptoms in 12 of 16 experimental studies (Pote, 2022).

Alongside participatory arts interventions, (such as clubs or classes delivered by artists or teachers), the *arts therapies*, which combine arts and psychotherapy, hold particular potential for treating CYPs with emotional distress. There are four arts therapy modalities: music therapy, art therapy, dance movement psychotherapy and dramatherapy; each of these are delivered by qualified therapists and many countries require state registration for these professions. Arts therapists are trained in both the use of their art form and, unlike in participatory arts interventions, also in psychotherapy. In addition to using creative methods, the arts therapies *also* offer participants: a therapeutic relationship, a safe and confidential space, elements of psychoeducation and space for self and emotional expression. In dramatherapy in particular, elements of drama, such as story, character and movement, are used as a means through which to process one's experience and to facilitate the therapeutic process (Klees, 2021). Although suitable for work with any age group, dramatherapy is particularly well suited to CYPs. The playful and creative nature of dramatherapy, and that it is frequently non‐verbal, means that CYPs can engage with the therapist, express themselves and process their lived experiences in an often enjoyable and helpful manner.

To date, various reviews of the arts therapies have been completed; while several research gaps have been identified, reviews have also reported promising findings (Freitas et al., 2022; Moula, 2020). Although generally, dramatherapy is the focus of less research than the other arts therapies, two key systematic reviews have been conducted. Feniger‐Schaal and Orkibi (2020) reviewed dramatherapy with people of all ages. While they determined that dramatherapy is an effective treatment for a number of populations, 46% of their studies related to developmental disability or cognitive impairment; mental health was not a primary focus. A review of dramatherapy with CYPs experiencing a broad range of psychosocial problems has also been conducted (Berghs et al., 2022). While this review included some studies related to mental health, studies also related to CYPs with conditions such as Asperger's syndrome, conduct disorder and learning disability; mental health was not a primary focus. A more focussed lens, such as the one taken in this review looking specifically at emotional distress, is required if one wishes to determine the effectiveness of dramatherapy interventions for common mental health conditions in CYPs.

As this review is, to our best knowledge, the first systematic review seeking to understand dramatherapy as a treatment specifically for emotional distress, the first two research questions sought to gather descriptive details of dramatherapy that is delivered with this aim:

Research Question 1 (RQ1) What are the population characteristics (such as age, gender and diagnosis) of children and adolescents who are engaged in dramatherapy due to emotional distress as reported in the literature?

Research Question 2 (RQ2) What are the intervention characteristics (such as length of sessions, number of sessions and setting) of dramatherapy for children and adolescents with emotional distress as reported in the literature?

The third question related to the benefits of dramatherapy:

Research Question 3 (RQ3) Where outcome data is available, what is the effect of dramatherapy on reducing emotional distress and improving the well‐being of children and adolescents? Where it is possible to calculate this, what is the range of effect sizes?

As identified by the research questions, this review concerns and includes dramatherapy interventions that are used in response to CYPs experience of emotional distress. Emotional distress is used throughout this review to characterise internalising mental health symptoms; these symptoms relate to one's internal emotional state and commonly include anxiety, depression and traumatic stress. A person's experience of emotional distress may or may not relate to a specific diagnosis or mental health condition and levels of distress may vary continuously. Furthermore, where a condition is diagnosed, this may occur alone or, may co‐occur alongside another condition. Papers were deemed suitable for inclusion in this review if the intended outcome of the work related to a reduction in symptoms of emotional distress. As such, papers related to broader mental health conditions (such as anorexia) and other conditions (such as autism spectrum disorder [ASD]) are included. Papers wherein participants had received no diagnosis but experienced elements of emotional distress and difficultly are also included.

## METHODS

This systematic review follows the Preferred Reporting Items for Systematic Review and Meta‐Analyses (PRISMA) guidelines as outlined by Moher et al. (2009). The protocol for this review was registered on PROSPERO (CRD42022310960).

### Search strategy

A total of seven electronic databases (PsychInfo, PubMed, Scopus, Web of Science, CINAHL, EMBASE and Cochrane) were identified as being suitable for use in this review. The patient/population, intervention, comparison and outcomes framework was used to identify relevant search terms and the strategy presented in Table 1 was employed.

**TABLE 1:** Search strategy employed in this review.

Dramatherap* OR	Child* OR	Mental Health OR
Drama therap* OR	Adolescen* OR	Mental illness OR
Drama psychotherap* OR	Youth OR	Mental well being OR
Theatre therap* OR	Teen* OR	Mental wellbeing OR
Arts therap* (drama) OR	Young pe* OR	Mental dis* OR
Creative arts therap* (drama) OR	Young adult*	Anxi* OR
Psychodrama*	Pubescent OR	Depress* OR
	Older child*	Schizophren* OR
AND		Bipolar* OR
	AND	Personality disorder* OR
		Trauma OR
		PTSD OR
		Eating disord*

The searches were completed, alongside database specific truncation and indexing terms, between 2^nd^–8^th^ March 2022. In addition, grey literature and three academic journals (Dramatherapy, Drama Therapy Review and Arts in Psychotherapy) were hand searched between 13^th^–15^th^ April 2022.

### Eligibility criteria

The eligibility criteria for this review were as follows: (a) peer‐reviewed articles which were published in English on any date, (b) the study sample included children and/or adolescents between the ages 8–18 years (or where the mean age of the sample fell in this range), (c) the study participants were reported to experience emotional distress (including participants with neurodiversity and learning disabilities), (d) the intervention was dramatherapy which was delivered either alone or in conjunction with another intervention, (e) the study comprised empirical research with quantitative and/or qualitative data. Studies that investigated the effects of drama clubs, lessons or groups and/or where the sessions were not led by an accredited therapist were excluded.

A lower age limit of 8 years was selected because research suggests that, at this age, children become able to reliably self‐report on various aspects of their health and well‐being (Riley, 2004). An upper age limit of 18 years was chosen because, in many countries, young people legally become adults and, crucially, move from child to adult healthcare services. Although two studies reported samples where the age range or mean age was unclear, additional information provided in the text allowed us to infer that the study samples met the age eligibility criterion; in one study the participants were listed as school age (Godfrey & Haythorne, 2013) and, for the second, participants were listed as ‘latency age’ (Haen & Brannon, 2002).

### Data extraction

The data extraction process was completed independently by two reviewers [EK] [MT] using a piloted extraction form. In addition, the TIDier checklist was completed by one reviewer [EK] to check the depth and suitability of the data extracted.

Extracted study characteristics were:

- Age of participants
- Gender or gender mix
- Information on the ethnicity of participants
- Information on the socioeconomic status of participants
- The presenting problem of the participants
- Exposure among participants to a specific set of circumstances (a traumatic event, being of refugee or adoptee status, etc)
- Primary outcome of the intervention
- Format (Group or 1‐1)
- Group size
- Setting (school, inpatient, etc)
- Number of sessions
- Frequency of sessions
- Duration of sessions
- Study design

### Quality & bias checking

Once identified, included studies were independently assessed for bias by two reviewers [EK] [MT]. As a range of study types were expected to be retrieved in this review, the Joanna Briggs Institute (JBI) Critical Appraisal Tools (2020) were selected for this process. The JBI contains a suite of tools which are specifically tailored to each study type and thus, suited this project well. Qualitative studies are typically evaluated on their descriptive quality and transparent reporting while quantitative evaluation focuses on the use of appropriate data and clear analysis. Following the independent screening, the two reviewers [EK] [MT] met to discuss, and agree upon, their ratings. No studies were excluded from this review as a result of bias.

### Data synthesis

RQ1 and RQ2 related to the descriptive characteristics of dramatherapy which are displayed in Table 2 and summarised in the text. As RQ3 sought to determine the effect of dramatherapy on emotional distress, relevant quantitative outcome measures (Table 3) were identified. Where possible, data relating specifically to internalising symptoms of emotional distress were extracted. High heterogeneity across outcome measures, participant characteristics and in the delivery of dramatherapy meant that meta‐analysis was not possible. Instead, where data were available, effect sizes, reflecting changes in pre‐to‐post intervention means were calculated using Cohen's *d* for intervention and control groups. Where data were missing, authors were contacted for further information. In two papers, by Hylton et al. (2019) and Rousseau et al. (2007) effect sizes were calculated using paired t‐scores and degrees of freedom as, following contact with the authors, the means and standard deviations were not available; calculating effect sizes in this way, may lead to inflated figures. In two studies (Hylton et al., 2019; Moula et al., 2020), dramatherapy was delivered as part of a wider study involving all arts therapy modalities; where this was the case, data relating only to the dramatherapy element of the study was extracted.

**TABLE 2:** Description of included studies; studies are arranged by design to aid in the synthesis.

	Study ID	Location				Participants						Intervention					Outcome Measures
		Country	Setting	Experimental condition	Control condition	No. of experimental participants	No. of control participants	Total participants	Age	Sex	Presenting problem	No. of sessions	Freq. of sessions	Duration of sessions	Format	Group size	
Randomised controlled trials	McArdle et al., 2002	UK	School	Dramatherapy	Adult‐led curriculum studies	60	62	122	11.35 years (mean)	Mixed	Participants were at risk of developing behavioural or emotional problems	12	Weekly	1 h	Group	8	TRF, YSR, MSCS, CBCL
	Rousseau et al., 2007	Canada	School	Dramatherapy (inc. Forum & playback theatre)	N/R	66	57	123	12–18 years	Mixed	Emotional & behavioural problems. Participants were recent immigrants & refugees to Canada	9	Weekly	75 min	Group	N/R	SDQ, SES, school performance based on GPA
	Moula, et al., 2020*	UK	School	Dramatherapy (alongside music, dance & art therapy)	Waitlist	DT: 7 total: 31	31	62	7–10 years	Mixed	Mild emotional & behavioural difficulties	8	Weekly	1 h	Group	6‐8	SDQ, EQ‐5‐DY, CORS
Quasi‐ experimental studies	Mackay et al., 1987	Canada	University drama studios	Dramatherapy	N/A	5	N/A	5	12–18 years	Female	Participants had all experienced sexual abuse	8	Weekly	4–5 h	Group	5	BDI, SCL‐90, TSBI, ASQ, SSQ, MCSDS
	Hylton et al., 2019	USA	School	Dramatherapy (alongside music & visual art therapy)	N/A	11	N/A	34	14–18 years	Mixed	Depression, anxiety, PTSD. Participants had experienced a school shooting	8	Four days per week for 2 weeks	3.5 h	Group	11	PHQ‐8, GAD‐7, CRTES, CPSS, PANAS
Mixed methods studies (quasi‐ experimental & qualitative)	Pellicciari et al., 2013	Italy	Inpatient eating disorder unit	Dramatherapy	N/A	15	N/A	15	14–19 years	Mixed	Anorexia nervosa	6–15 (average of 10)	Weekly	N/R	Group	15	TAS‐20, SAFA O, SAFA D
	McLachlan & Laletin, 2015	UK	Outpatient CAMHS	Dramatherapy with mindfulness	N/A	4	N/A	4	15 & 16 years	Female	Anxiety, low mood & body dysmorphic disorder	5	Weekly	N/R	Group	4	CAMM, RCADS, ESQ
Qualitative studies	Godfrey & Haythorne, 2013	UK	School	Dramatherapy	N/A	42 parents	N/A	42 parents	“School age”	Mixed	Autism spectrum disorder	N/R	Weekly	N/R	N/R	N/R	Thematic analysis of parent feedback
	Jarman, 2014	UK	CAMHS	Dramatherapy	N/A	4	N/A	4	7–9 years	Male	Trauma. Participants had witnessed domestic abuse	15	Weekly	N/R	Group	4	Thematic analysis of semi‐structured interviews
	Burch et al., 2019	USA	School	Dramatherapy (inc. Therapeutic theatre)	N/A	6	N/A	6	16.2 years (mean)	Mixed	Depression, anxiety, stress. Participants were at‐risk of high school dropout	N/R	Weekly	N/R	Group	6	BASC‐3, RS (but these are not reported in this paper)
	Moula, 2021*	UK	School	Dramatherapy (alongside music, dance & art therapy)	Waitlist	DT: 7 total: 31	31	62	7–10 years	Mixed	Mild emotional & behavioural difficulties	8	Weekly	1 h	Group	6‐8	Thematic analysis of semi‐structured interviews
Case studies	Barsky & Mozenter, 1976	USA	Not reported	Dramatherapy	N/A	7	N/A	7	8–10 years	Mixed	Emotional and behavioural difficulties	N/R	N/R	N/R	Group	7	N/A
	Haen & Brannon, 2002	USA	Residential treatment facility	Dramatherapy	N/A	Not reported	N/A	N/R	“Latency age”	Male	Mood disorder, depression, psychotic disorders, PTSD	N/R	Weekly	N/R	Group	N/R	N/A
											Participants were in residential care						
	Millbrook, 2019	UK	Online	Dramatherapy	N/A	2	N/A	2	15 years	Mixed	Depression, anxiety, OCD. Participants had self‐excluded from mainstream education	N/R	N/R	N/R	One to one	N/A	N/A
	Moore, 2019	UK	Home and school	Dramatherapy (life story therapy)	N/A	3	N/A	3	7–13 years	Mixed	PTSD, foetal alcohol effect, ASD. Participants were looked after children.	6–53	N/R	N/R	One to one (plus carers)	1	SDQ, boxall profile (not reported in this paper)

Abbreviations: ASD, autism spectrum disorder; ASQ, attributional style questionnaire; CAMM, child and adolescent mindfulness measure; CBCL, child behavior checklist; CORS, child outcome rating scale; DT, dramatherapy; ESQ, experience of service questionnaire; GPA, grade point average; MCSDS, Marlowe‐Crowne social desirability scale; MSCS, multidimensional self concept scale; OCD, obsessive–compulsive disorder; PANAS, positive and negative affect schedule; RCADS, revised child anxiety and depression scale; RS, resilience scale; SAFA, self administrated psychiatric scales for children and adolescents; SES, self esteem scale; SSQ, social support questionnaire; TRF, teacher report form.

* Studies were derived from the same investigation.

**TABLE 3:** Outcome measures and effect sizes relating to emotional distress ( = large,  = medium to large,  = small to medium,  = negligible to small).

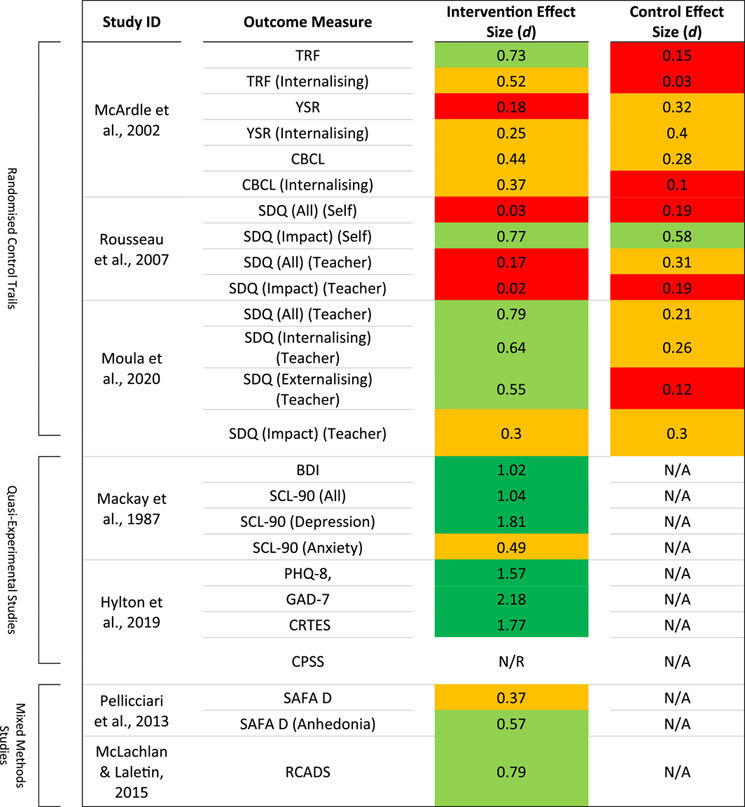

## RESULTS

**FIGURE 1:**
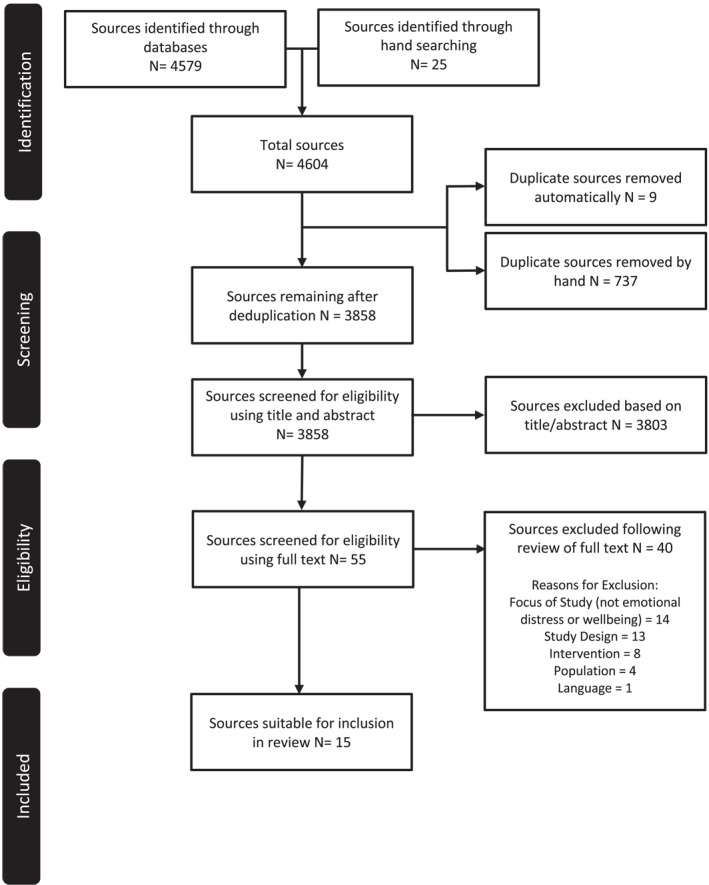
Systematic search process reported according to Preferred Reporting Items for Systematic Review and Meta‐Analyses (PRISMA) guidelines (Moher et al., 2009).

The search process (using databases and hand searches) yielded a total of 4604 results, 746 of these were duplicates which were subsequently removed. A total of 3858 titles and abstracts and 55 full texts were screened by the lead reviewer [EK]. Papers in the full text screening stage were discussed with a second reviewer [JL] and decisions for inclusion or exclusion were agreed upon. At the end of the screening process, a total of 15 articles were identified as suitable for inclusion in this review. A detailed PRISMA flow diagram (Moher et al., 2009), outlining both the stages and reasons for exclusion, is provided in Figure 1.

### Study characteristics

Of the 15 included studies (Table 2), eight were published in the UK, four were published in the US, two were published in Canada and the remaining study was published in Italy. The date of the studies ranged from 1976 to 2021 but only two studies (published in 1976 & 1987) were published before 2000. Three studies were published between 2000 and 2010 and the majority, 10 studies, had been published since 2010.

Five of the included studies (33.3%) were purely quantitative and the authors had collected pre‐ and post‐ intervention data. Of the five quantitative studies, just three studies (20%) utilised a controlled condition; no other studies used in this review (80%) were controlled. Two studies (13.3%) were mixed methods and the authors collected both quantitative and qualitative data. Four studies (26.7%) were case studies. Four studies (26.7%) were qualitative and presented the analysis of feedback or interviews following a dramatherapy intervention. Two of the 15 papers (Moula, 2021; Moula et al., 2020) were derived from data gathered from the same intervention; one of these papers reviewed the intervention quantitatively while the other did so qualitatively. Therefore, while there were 15 papers, data from 14 independent samples were reported on.

The total number of participants in each study ranged from two to 123 across all study types. In the RCTs, the total participants ranged from 62 to 123 while in the quasi‐experimental studies, the number ranged from five to 34. In the mixed methods studies, the number of participants was between four and 15 and in the qualitative studies, the numbers were between four and 62. Finally, for the case studies, the number of participants ranged between two and seven.

### RQ1: Population characteristics

Across all studies, the total number of CYPs who received a dramatherapy intervention was 190; the number of CYPs who received a control intervention was 150; one paper involved 42 parents.

*Age:* The age of the CYPs ranged from 7 to 18 years. Of the 15 studies, four studies involved children who were aged 11 or under. Eight of the studies involved adolescents, who were between 12 and 18 years old, and one study listed their participants as being between 5 and 12 years old thus, involving both children and young adolescents. Two studies (Godfrey & Haythorne, 2013; Haen & Brannon, 2002) reported unclear age ranges.

*Gender:* 11 studies involved a mixture of male, female and non‐binary participants. Of the remaining four studies, two were female‐only groups and two were male‐only groups.

*Presenting Problem:* While all 15 studies aimed to alleviate emotional distress, only two studies reported homogeneous samples based on clinical diagnosis: one of these involved participants with anorexia (Pellicciari et al., 2013) and one involved participants with autism spectrum disorder (Godfrey & Haythorne, 2013). In contrast, six studies reported dramatherapy as being delivered to groups containing a range of diagnoses including depression, anxiety, body dysmorphic disorder and obsessive‐compulsive disorder. Where mixed diagnoses were present, participants were often grouped in relation to a shared experience or circumstance. Such circumstances included: having experienced a school shooting (Hylton et al., 2019), having self‐excluded from education (Millbrook, 2019), being at risk of high school drop‐out (Burch et al., 2019) or living in residential care (Haen & Brannon, 2002; Moore, 2019). Five studies listed their participants as experiencing, or being at risk of experiencing emotional and behavioural problems; one of these involved participants who were recent immigrants or refugees (Rousseau et al., 2007). Trauma was listed in one study, wherein the participants had all witnessed domestic violence (Jarman, 2014) and one study did not give a clinical diagnosis, but all participants had experienced sexual abuse (Mackay et al., 1987).

### RQ2: Intervention characteristics

*Setting:* This varied across the 15 studies. In seven studies, the intervention took place in schools with primary and secondary schools accounting for three studies each and the remaining study (Godfrey & Haythorne, 2013) taking place across both. Two studies took place in outpatient Child and Adolescent Mental Health Services (CAMHS) (Jarman, 2014; McLachlan & Laletin, 2015), and one was in an inpatient eating disorder unit (Pellicciari et al., 2013). Other settings described in the studies were: the home, ‘a residential treatment facility’ (Haen & Brannon, 2002), a university building (Mackay et al., 1987) and an online setting (Millbrook, 2019); each of which were named in one study each. In one study, the setting of the intervention was unclear (Barsky & Mozenter, 1976).

*Delivery:* The majority of studies related to group interventions with 13 of 15 reporting that a group was their mode of delivery. Just two studies offered 1‐1 dramatherapy; one of these (Moore, 2019) also involved foster/adoptive parents in the process (although it should be noted that parents were supporting the child's process rather than receiving therapy themselves).

*Number of Sessions:* 14 studies delivered between five and 12 sessions. One study (Moore, 2019) reported that up to 53 sessions were given to one participant and their family. In eight of the studies, the dramatherapy took place weekly while one study (Hylton et al., 2019) took a more intensive approach and offered dramatherapy for 4 days per week across a 2‐week period; it should be noted that this latter study took place in a school summer camp and thus, the design and delivery may have been determined by those conditions. In six studies, the frequency of sessions was not reported.

*Duration:* Three papers (McArdle et al., 2002; Moula, 2021; Moula et al., 2020) reported that their sessions ran for 1 hour and one stated 75 min (Rousseau et al., 2007). Significantly longer sessions were reported in two studies, with one stating 3.5 h (Hylton et al., 2019) and another, 4–5 h per session (Mackay et al., 1987). Nine studies did not report data relating to session duration.

### RQ3: Outcomes and effect

Of the 15 studies included in this review, seven studies reported quantitative data that is relevant to RQ3.

### Measures of emotional distress

Within the seven studies reporting quantitative data, 12 measures relating to emotional distress were reported on; these measures are displayed in Table 3. Across the measures, pre‐to‐post intervention effect sizes ranged from *d* = 0.17 to *d* > 2 reflecting a wide range of therapeutic improvements.

*Trauma:* The two studies producing the largest effect sizes were by Hylton et al. (2019) and Mackay et al. (1987). However, these studies were quasi‐experimental studies without a control condition. These studies were similar in that they both provided dramatherapy to adolescents who had experienced a significant trauma. For Hylton et al. (2019), participants (mean age 14.7 years) had experienced a school shooting and, for Mackay et al. (1987), participants (mean age 15.4 years) had experienced sexual abuse. The delivery of the dramatherapy was also comparable as sessions were delivered in a group format, for eight sessions and via a longer session (>3 h) than most other studies. Both studies reported the largest effects on depression measures. For Hylton et al. (2019), data collected via the PHQ‐8 generated an effect size of *d* = 1.57. For Mackay et al. (1987), data collected via the Beck Depression Inventory (Beck et al., 1987) generated an effect size of *d* = 1.02 and depression data for the Symptom Checklist‐90 (SCL‐90) (Vaurio, 2011) generated *d* = 1.81. Interestingly, effect sizes related to anxiety measures in both papers were not similar; Hylton et al. (2019), reported a large effect size of *d* = 2.18 on data collected via the GAD‐7 but for, Mackay et al. (1987), anxiety data from the SCL‐90 generated a small to medium effect of *d* = 0.49. Hylton et al. (2019), also employed outcome measures related to participants' response to trauma and was the only paper to do so. Both the Child's Reaction to Traumatic Events Scale (CRTES) (Jones, 1996) and the Child PTSD Symptom Scale (CPSS) (Foa et al., 2001) were listed in the methods of this study, however, CPSS data was not published. The CRTES data (*p* = 0.023) generated a large effect size (*d* = 1.77) between the pre‐ and post‐intervention.

*Clinical Settings:* No further studies reported large effects, however, medium to large effect sizes were reported in several papers. Two studies (both uncontrolled), which reported such effects, were by McLachlan and Laletin (2015) and Pellicciari et al. (2013). These studies both took place in clinical mental health settings. In McLachlan and Laletin's (2015) study, five, weekly, dramatherapy and mindfulness sessions were delivered in CAMHS. For Pellicciari et al. (2013), weekly dramatherapy was delivered for 15 weeks (participants attended an average of 10 sessions) to 15 adolescent participants who were hospitalised with anorexia. Both studies involved (primarily) adolescent females; all four participants in McLachlan and Laletin's (2015) study and 14 of 15 participants in Pellicciari et al.’s (2013) were female. Medium to large effect sizes were reported in both studies; via the Revised Child Anxiety and Depression Scale (Chorpita et al., 2000), McLachlan and Laletin (2015) reported an effect size of *d* = 0.79. It should be noted that high heterogeneity was highlighted in symptom reductions among the four participants in this sample; while one participant had a “meaningful reduction in symptoms” (McLachlan & Laletin, 2015, p. 84), two participants experienced minimal reduction and one, a very slight increase. For Pellicciari et al. (2013), a medium to large effect size (*d* = 0.57) was seen in data related to anhedonia, as collected via the Scale for Evaluation of Depression (self administrated psychiatric scales for children and adolescents [SAFA]‐D) (Franzoni et al., 2009), however, effect size for the full measure of depression was small to medium (*d* = 0.37).

*School Settings:* Five studies aimed to reduce symptoms of emotional and behavioural difficulties in participants; three of these reported quantitative data which could be used to calculate an effect size. One study, which did so, was a randomised controlled trial by McArdle et al. (2002). In this study, dramatherapy was delivered in primary, middle and secondary schools (mean age 11.4 years) in an area of economic deprivation. Another study which took place in schools, was by Moula et al. (2020). This study investigated group arts therapies on the quality of life and wellbeing of children aged 7–10 years who had mild emotional and behavioural difficulties. Both of these studies were UK based and ran their intervention for between eight and 12 weeks. Medium to large effects were seen in these studies on data that had been reported by the participants' class teachers. For McArdle et al. (2002) internalising data collected via the Teacher Report Form generated a medium to large effect size (*d* = 0.52) following the dramatherapy intervention. For the curriculum studies control group, this figure was just *d* = 0.03. Interestingly, smaller effect sizes were seen in both self‐ and parent‐reported data (*d* = 0.25 and *d* = 0.37 respectively) than in the teacher‐reported data. This pattern, of teacher‐reported data generating a larger effect size than self‐ or parent‐reported data, was sustained at 1 year follow up. In Moula et al.’s (2020) study, teacher‐reported data also generated a medium to large effect size. Internalising data were extracted from the Strengths and Difficulties Questionnaire (SDQ) (Muris et al., 2003) and the effect size following the dramatherapy group was *d* = 0.64; for the control group, this figure was small to medium (*d* = 0.26).

In contrast to these promising findings, a third study which also sought to reduce symptoms of emotional and behavioural difficulties was by Rousseau et al. (2007). Unlike McArdle et al.’s (2002) and Moula et al.’s (2020) studies which focused on children, the participants in this study were adolescents aged between 11 and 18 years; they also were recent immigrants and refugees to Canada. In this study, 10 weekly, 75 min dramatherapy sessions were delivered as part of school‐based integration workshops. Both the teacher‐ and self‐report SDQ were reported on in this study and the effect sizes were *d* = 0.17 and *d* = 0.03 respectively. While the teacher‐report SDQ was *slightly* higher, the two figures were considered small and insignificant, respectively. Interestingly, data relating to the SDQ impact supplement yielded a medium to large effect size (*d* = 0.77) for self‐report data. However, this figure was insignificant for teacher‐reported data (*d* = 0.02).

### Other measures

Studies also reported on a range of measures which were not directly related to emotional distress but related generally to participant health or wellbeing. Although not of primary interest for this review, data relating to externalising symptoms, behaviour, self‐esteem, alexithymia and more are reported on in Table 4.

**TABLE 4:** Outcome measures and effect sizes not relating to emotional distress ( = large,  = medium to large,  = small to medium,  = negligible to small).

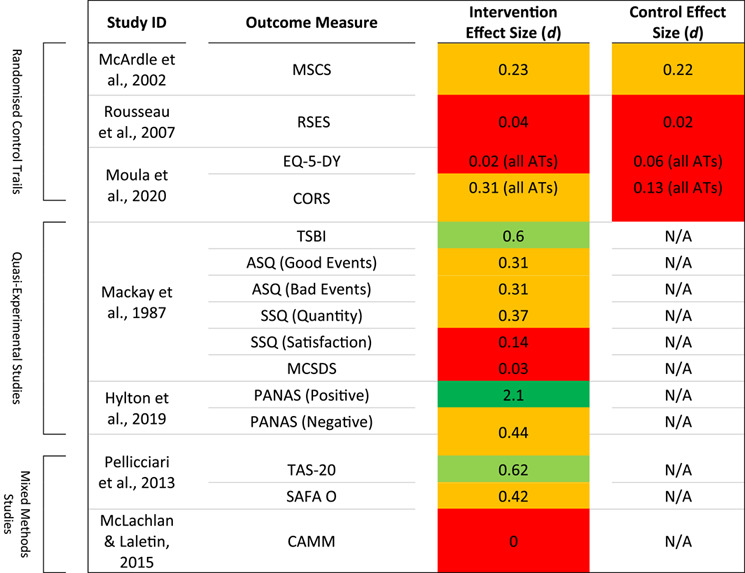

### Risk of bias

**FIGURE 2:**
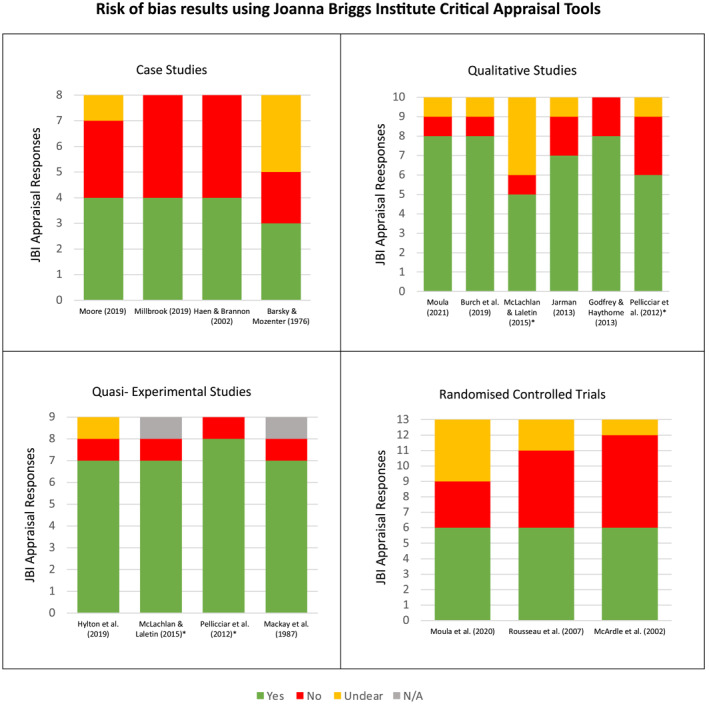
Results of the Joanna Briggs Institute (JBI) Critical Appraisal *Studies were mixed methods and were appraised as per each method used.

Figure 2 shows the risk of bias in each study following measurement against the JBI (2020) critical appraisal tools. None of the studies were without risk of bias however, no studies were excluded on this basis.

*Quantitative studies:* The three RCTs (McArdle et al., 2002; Moula et al., 2020; Rousseau et al., 2007) were all deemed to be of similar quality. Of particular strength were the selection and application of appropriate outcome measures and the completion of suitable statistical analyses. While none of the RCTs statistically addressed baseline differences between their intervention and control groups, all three studies did consider potential baseline differences in emotional distress either by controlling for these in their analysis (McArdle et al., 2002; Rousseau et al., 2007) or via pre‐ to post‐intervention change scores (Moula et al., 2020). A particular weakness of the RCTs related to the blindness of outcome assessors; in one study (Moula et al., 2020), it was unclear if assessors were blinded to participants intervention status and, in two studies, assessors were not blind (McArdle et al., 2002; Rousseau et al., 2007). It should also be noted that true randomisation, a key feature of bias reduction, was not uniformly employed across the RCTs. For example, in Rousseau et al.’s (2007) study, it is unclear how allocation to the control or intervention arm was conducted and for McArdle et al. (2002), teachers placed children into groups before each group was randomly allocated to either intervention or control. Positively, in Moula et al.’s (2020) study, a random number generator was employed for the group allocation (although if too many participants were of the same gender, the groups were altered for balance thus, potentially introducing some bias). A final point with regards to bias in the RCTs, is that there was limited information in all studies with regards to the identical treatment of the intervention and control groups.

The quasi‐experimental studies (two of which were mixed methods) were deemed to be methodologically strong with each of the four studies (Hylton et al., 2019; Mackay et al., 1987; McLachlan & Laletin, 2015; Pellicciari et al., 2013) only lacking a control group. These studies were all considered to have used multiple, reliable outcome measures and to have completed appropriate data analyses. Although not measured by the JBI (2020), it should be noted, that potential demographic confounders were not considered when evaluating the effect of the interventions. Albeit not related to effect, Hylton et al. (2019) did investigate completers versus non‐completers with regards to race, age, school attended and symptoms of distress among other variables; these characteristics were found to have no impact on participants' completion status. The quasi‐experimental studies were all deemed to be clear in terms of ‘cause and effect’ (Joanna Briggs Institute, 2020) (i.e. the symptom change followed the dramatherapy) and follow up was complete or adequately described. It should be noted that sample size was not interrogated by the JBI and this, along with a lack of control, was the predominant methodological weakness in these studies.

*Qualitative studies:* The qualitative studies (two of which were mixed methods) were somewhat more varied in quality, however, all six studies scored positively in relation to the methods selected, the analysis and interpretation of the data and the conclusions drawn by the authors. The voices of the participants were deemed to be adequately represented in all but one study; in Jarman (2014), due to the lack of direct quotations, it was deemed to be unclear whether the conclusions drawn were an appropriate representation of the participants' voices. Just two qualitative studies, those by Godfrey and Haythorne (2013) and by Jarman (2014), scored positively in relation to congruence between the stated philosophical perspective and the research methodology selected; the remaining four studies did not locate the research in this way. Similarly, in just two studies (Burch et al., 2019; Moula et al., 2020) was the influence of the researcher on the research adequately addressed.

*Case studies:* The studies with the highest risk of bias were the case studies. All four case studies scored positively in relation to the description of the intervention in question, however, just three (Haen & Brannon, 2002; Millbrook, 2019; Moore, 2019) gave a sufficient description of the patient's demographic characteristics and clinical condition. Only Moore (2019) supported this with the patient's clinical history. None of the studies gave detail regarding the post‐intervention clinical condition or diagnostic tests used. Take away lessons were provided in three of the four studies with just Moore (2019) overlooking such information. Although methodological improvements could be made to these studies, the varied results may be due to the nature of case studies in dramatherapy and what it is they seek to explore; for example, Haen and Brannon (2002) investigated the roles commonly taken by participants in a dramatherapy group, rather than the clinical outcomes of such work.

## DISCUSSION

Dramatherapy interventions for CYPs experiencing emotional distress have not previously been systematically reviewed; as such, this review sought to determine the nature, scope and effect of dramatherapy which is currently delivered for this purpose. RQ1 of this review sought to determine the characteristics of participants who are engaged in dramatherapy as a treatment for emotional distress. The participants involved were largely heterogeneous and no ‘typical’ dramatherapy participant was determinable from the findings. Age parameters, of 8–18 years, were pre‐determined for this review and the age of participants extended to these parameters. This suggests that dramatherapy is currently delivered to CYPs of ranging ages and the practice is not necessarily perceived to be suitable only for younger or older participants. The gender of participants was also varied; papers involved both mixed and single sex groups in relatively equal numbers, further suggesting range and variability in the practice. The wide range of participant characteristics in this review is reflected in the reviews of other arts therapies; Carr and Wigram's (2009) review of children's music therapy involved participants ranging from 5 to 16 years while Moula's (2020) review of art therapy in primary schools covered the full range of this age group (5–12 years). A lack of reporting regarding participant characteristics was present in a handful of studies. For example, in two studies (Godfrey & Haythorne, 2013; Haen & Brannon, 2002), the age of participants was unclear. Complete data relating to participant characteristics should be included in future research studies; the omission of such data makes it challenging to draw conclusions relating to the effect and suitability of dramatherapy interventions.

The range of diagnoses and presenting problems reported in the papers in this review is also notable. In six studies (40%) participants were placed in mixed diagnoses groups with common factors being among the setting they are drawn from, their age or a shared experience. Not only does this suggest that dramatherapy is currently viewed as a suitable treatment for a range of clinical needs and severities but it also supports the theory of group psychotherapy as identified by Yalom. To cultivate the 11 therapeutic factors, such as universality and interpersonal learning, a group must contain both heterogeneity and homogeneity (Yalom, 1985). It should also be considered for this review that the clinical make up of groups may be a product of the setting in which the dramatherapy took place; school settings, for example, may produce groups with varied or no diagnoses whereas clinical settings may lend themselves more to diagnostic homogeneity. In four studies (26%), dramatherapy was delivered to groups wherein the participants had experienced a shared traumatic experience such as abuse or displacement. Shared adversity is also the focus of a recent review of art therapy (Annous et al., 2022) for traumatized refugee CYPs. The review, within which all studies relate to groups, concludes that art therapy is a “promising treatment approach” in this area (Annous et al., 2022, p. 1). These findings may suggest that a shared or common experience within a group is a useful component of effective arts therapy and more research in the area is warranted.

This review also sought to explore intervention characteristics (RQ2). Data relating to the setting, mode of delivery, number of sessions and duration of sessions was extracted from all studies. Of particular note was that 13 studies involved group dramatherapy and just two studies involved one‐to‐one provision (Millbrook, 2019; Moore, 2019). This reflects findings from a large review of dramatherapy by Feniger‐Schaal and Orkibi (2020) wherein, of 24 dramatherapy interventions reviewed, just two related to one‐to‐one provision. While this may suggest that dramatherapy is more commonly delivered in a group format, it may also be true that one‐to‐one dramatherapy is simply not reported on to the same degree and one should remain cautious of these findings. It is also of note, regarding intervention characteristics, that just under half of the studies (seven) took place in schools; this finding aligns with data from the British Association of Dramatherapists (2022) wherein it was estimated that close to 40% of UK dramatherapists are employed in school settings.

Providing dramatherapy in schools may offer a number of advantages including fewer barriers to access and less time spent away from education when receiving treatment. School based dramatherapy may also be delivered to children whose symptoms do not meet a clinical threshold. Providing dramatherapy at this stage may prevent worsening and may reduce the need for referral to clinical services; further research into this area is warranted. The studies involved in this review typically offered between five and 12 sessions of dramatherapy which reflects the typical number of sessions offered in NHS talking therapies (NHS Digital, 2022). However, research regarding the ideal number of sessions for effective change in dramatherapy is also warranted. Finally, the duration of sessions was not reported on in the majority of studies with nine studies omitting this data entirely. This data would be useful in determining the dosage of effective dramatherapy and would contribute toward manualising practice.

RQ3 of this review sought to determine the effect of dramatherapy on symptoms of emotional distress, however, as just seven studies reported quantitative data relating to symptom reduction and effectiveness, one must be cautious when drawing conclusions from this sample. The largest effects were seen in two quasi‐experimental studies which sought to address adolescent participants' trauma. Both Hylton et al.’s (2019) and Mackay et al.’s (1987) studies saw large effect sizes in data relating to symptoms of depression, however, these studies did not have a control and may contain bias. Although slightly smaller, notable effect sizes were seen in McLachlan and Laletin's (2015) and Pellicciari et al.’s (2013) studies; these studies both took place in clinical mental health settings where the participants involved are likely to have met a high symptom threshold. For McLachlan and Laletin's (2015), medium to large effect sizes were seen in relation to anxiety and depression symptoms while, for Pellicciari et al. (2013), for depression, a small to medium effect size was seen; in data relating specifically to anhedonia, the effect size was medium to large. Like Hylton et al.’s (2019) and Mackay et al.'s studies, McLachlan and Laletin's (2015) and Pellicciari et al.’s (2013) studies were also uncontrolled and thus, may contain bias. Alongside a lack of control, small sample sizes, such as four participants in McLachlan and Laletin's (2015) study and five participants in Mackay et al.’s (1987) study also mean that findings are suggestive at best. Larger sample sizes and robust control conditions should be a priority for future research in this area. Although firm conclusions cannot be drawn, the effect sizes discussed here may suggest that dramatherapy is an effective treatment for higher level symptoms of emotional distress or following significant trauma. The use of trauma focused outcome measures in future research is encouraged; this would allow for investigation specifically into the effect of dramatherapy on trauma symptoms.

A range of effect sizes were seen in studies which took place in schools and which sought to address participants' emotional and behavioural difficulties. Three school‐based studies (McArdle et al., 2002; Moula et al., 2020; Rousseau et al., 2007), all of which were RCTs, reported quantitative data relating to the effectiveness of their intervention. In both McArdle et al. (2002) and Moula, et al.'s (2020) studies, wherein the intervention took place in primary schools, medium to large effects were seen in teacher‐reported data; this was higher than effect sizes derived from self‐ or parent‐reported data in these studies. Albeit smaller, teacher‐reported data was *also* higher than self‐report in Rousseau et al.’s (2007) study wherein dramatherapy was delivered to refugee adolescents in secondary school. Notwithstanding the small sample, this data suggests that, when seeking to address emotional and behavioural difficulties, the positive impact of dramatherapy is experienced by teachers and that positive change is most prominent in the school environment; more research into this finding is warranted. Although these RCTs did employ a control group, methodological limitations, such as group allocation or blindness were still present. It was also unclear in all studies if the treatment and control groups were treated identically bar the intervention in question; the studies could have been further improved if clarity was given in relation to these areas. While these three studies suggest that dramatherapy is having a positive effect on emotional and behavioural symptoms when delivered in weekly groups in schools, a small number of studies, methodological limitations and varied results mean that it is not clear exactly what is contributing to this effect; further, more robust, research into this area is warranted.

Pertaining to the ranging effect sizes, to the relatively small sample sizes and to methodological limitations among the papers in this review, it is difficult to draw conclusions for dramatherapy as an overall treatment for emotional distress. The effect sizes seen throughout this review suggest that there is clinical potential for dramatherapy in a variety of settings but further research is required to explore the characteristics of dramatherapy which may lead to such effects.

### Limitations and recommendations for future research

This systematic review has a number of limitations, the first of which is in relation to study design. As it was not expected that a great number of studies would be suitable for inclusion in this review, all study designs were included; additionally, no studies were excluded from this review based on their quality. The inclusion of case studies, wherein no effectiveness data was collected and which, in dramatherapy, often contain the therapists' opinion in place of empirical data, limits the findings of this review, particularly in relation to RQ3. Another limitation of this review relates to the extent to which all relevant studies were able to be identified in the searches. For example, papers were required to be written in English as the review team did not have the financial resources required to utilise translation services. In addition, restrictions also were placed on the peer review status of the papers and, although this decision was taken to ensure the robustness of the included studies, relevant studies published by other means may have been excluded. The age parameters selected may also limit the generalisability of this review and findings may be less applicable to young adults (aged 18–24 years) or to pre‐adolescent children (aged under 8 years). However, the parameters were determined based on pre‐existing research and in order to reflect the age at which CYPs, in most countries, become legal adults. The number of studies in this review is also a limitation; the searches retrieved only seven studies with quantitative data that could be used to answer RQ3. Although containing quantitative data, these studies varied in design, sample composition and sample size while also using a wide variety of outcome measures; such heterogeneity made it difficult to draw patterns relating to the factors that modulate effect size. This review is also limited in that it did not seek to extract data relating to adherence or explore the content of the dramatherapy interventions it reviewed; extracting and evaluating this information may enable scrutiny in relation to dramatherapy content and subsequent outcomes.

In light of both the above limitations and those discussed throughout this review, it is recommended that future research focuses primarily on methodological robustness. The effect of dramatherapy should be investigated using rigorous control conditions and larger sample sizes determined via appropriate power calculations. Beyond methodology, future research may wish to explore the prevalence and effect of one‐to‐one dramatherapy or the perception of change between CYPs, their parents and teachers following a dramatherapy intervention. In addition, the ideal number of sessions required for psychotherapeutic benefit or symptom reduction of mental illness should also be investigated. Related work has begun in music therapy (Gold et al., 2009) and investigating this area with regards to dramatherapy with CYPs would be of benefit for both policy and practice alike. Finally, details relating to the content of dramatherapy sessions and the active ingredients required for positive change should also be investigated; researching this area may lead to useful and applicable clinical findings.

## CONCLUSION

The field of dramatherapy has a small but developing evidence base. This systematic review has revealed that, while some research exists, methodological improvements to research in this field are needed. It has also revealed the scope of dramatherapy and the versatility of this practice as a treatment for emotional distress. The findings suggest that dramatherapy has been studied mostly as a group intervention (87%) for adolescents (53%) however, it is used with CYPs of all ages, in a variety of settings and for a range of different clinical and emotional needs. The effect of dramatherapy was also reviewed. Although the sample was small, findings suggest that dramatherapy may be a suitable treatment for severe clinical symptoms and when used in school settings for early intervention; these findings may have clinical relevance and may impact upon policy in the future. As dramatherapy research is in relative infancy, future studies in this field may wish to go beyond questions of effect (Skivington et al., 2021). Robust research leading to knowledge in relation to adherence, implementation, cost effectiveness, uptake and the scalability of dramatherapy, would be of great value to this practice going forward.

## AUTHOR CONTRIBUTION

**Eleanor Keiller:** Conceptualization, Data curation, Formal analysis, Investigation, Methodology, Project administration, Resources, Software, Visualization, Writing – original draft, Writing – review & editing. **Megan Tjasink:** Formal analysis, Investigation, Validation. **Jane Bourne:** Writing – review & editing. **Dennis Ougrin:** Writing – review & editing. **Catherine Elizabeth Carr:** Supervision, Writing – review & editing. **Jennifer Y. F. Lau:** Supervision, Writing – review & editing.

## CONFLICTS OF INTEREST

Eleanor Keiller is a member of British Association of Dramatherapists' Research Subcommittee and conducts this role on a volunteer basis. The remaining authors of this paper have declared that they have no competing or potential conflicts of interest.

## ETHICS STATEMENT

As it is a systematic review, meaning that no primary data was collected, the authors did not seek ethical approval for this study.

## Data Availability

The effect sizes calculated for this review were calculated using the data available in the included studies or following direct contact with the study author.
